# A RasGAP SH3 Peptide Aptamer Inhibits RasGAP-Aurora Interaction and Induces Caspase-Independent Tumor Cell Death

**DOI:** 10.1371/journal.pone.0002902

**Published:** 2008-08-06

**Authors:** Perayot Pamonsinlapatham, Réda Hadj-Slimane, Françoise Raynaud, Marc Bickle, Claudine Corneloup, Audrey Barthelaix, Yves Lepelletier, Perrine Mercier, Matthieu Schapira, Jérôme Samson, Anne-Laure Mathieu, Nicolas Hugo, Olivier Moncorgé, Ivan Mikaelian, Sylvie Dufour, Christiane Garbay, Pierre Colas

**Affiliations:** 1 Université Paris Descartes, UFR Biomédicale, Laboratoire de Pharmacochimie Moléculaire et Cellulaire, INSERM U648, Paris, France; 2 Aptanomics, Lyon, France; 3 Université Paris Descartes, Hôpital Necker, CNRS UMR 8147, Paris, France; 4 UMR CNRS 144, Institut Curie, 26, Paris, France; Universität Heidelberg, Germany

## Abstract

The Ras GTPase-activating protein RasGAP catalyzes the conversion of active GTP-bound Ras into inactive GDP-bound Ras. However, RasGAP also acts as a positive effector of Ras and exerts an anti-apoptotic activity that is independent of its GAP function and that involves its SH3 (Src homology) domain. We used a combinatorial peptide aptamer approach to select a collection of RasGAP SH3 specific ligands. We mapped the peptide aptamer binding sites by performing yeast two-hybrid mating assays against a panel of RasGAP SH3 mutants. We examined the biological activity of a peptide aptamer targeting a pocket delineated by residues D295/7, L313 and W317. This aptamer shows a caspase-independent cytotoxic activity on tumor cell lines. It disrupts the interaction between RasGAP and Aurora B kinase. This work identifies the above-mentioned pocket as an interesting therapeutic target to pursue and points its cognate peptide aptamer as a promising guide to discover RasGAP small-molecule drug candidates.

## Introduction

The Ras signaling pathway plays a pivotal role in relaying numerous extracellular signals to various intracellular regulatory networks that control cell proliferation, differentiation and apoptosis. Ras switches between an active (GTP-bound) and an inactive (GDP-bound) state, which are produced by guanine exchange factors such as Sos and GTPase-activating proteins (GAP) such as RasGAP or neurofibromin, respectively [1].

**Figure 1:**
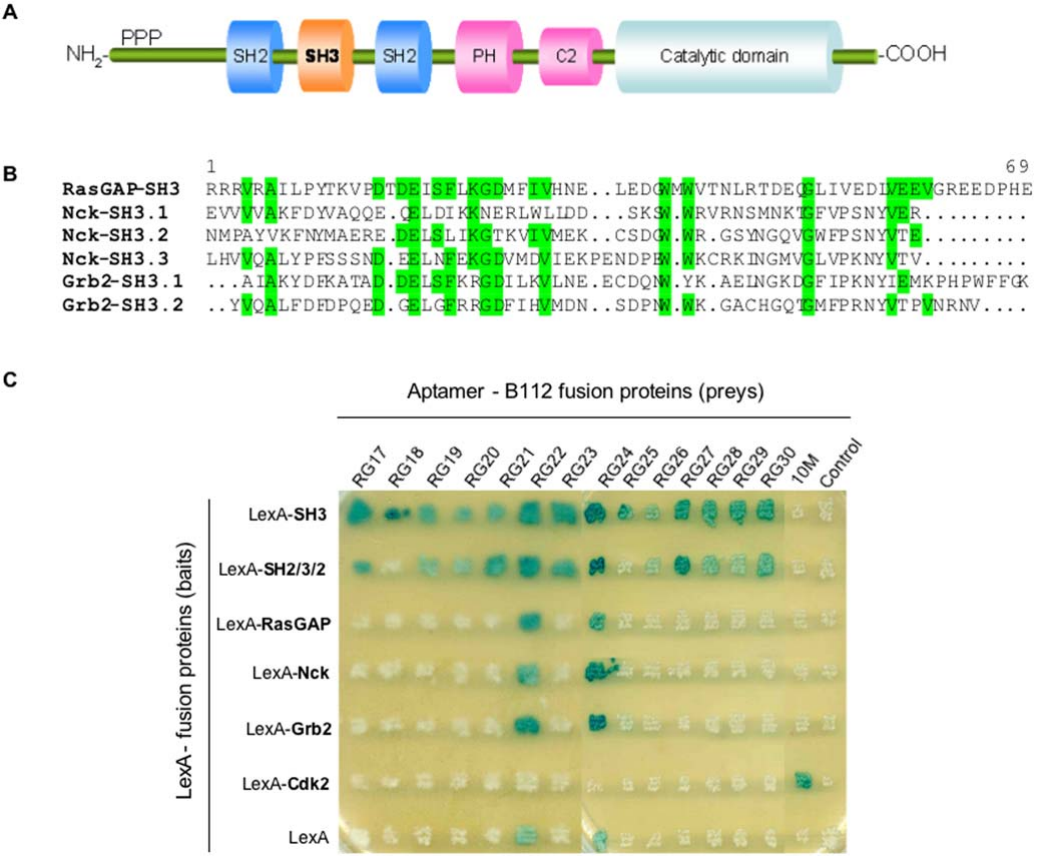
RasGAP SH3 peptide aptamer binding specificity. (A) Protein domain mapping of the RasGAP protein. PPP: Proline-rich region; PH: Pleckstrin homology domain; C2: pKC-conserved region 2. (B) Sequence alignment of RasGAP, Nck, and Grb2 SH3 domains. Residues that are conserved between at least two proteins are highlighted in green. (C) Yeast two-hybrid interaction mating assay. LexA fusion proteins (“baits”) were expressed in EGY191α yeast containing a 8 *lexAop-lacZ* reporter gene. Control and selected peptide aptamers (fused to the B112 activation domain) were expressed in EGY42a yeast. Strains were mated and diploids were grown on X-gal-containing solid medium. 10M is a Cdk2 peptide aptamer and Control is a peptide aptamer randomly picked from the library.

Initially, RasGAP has been identified as the main negative regulator of Ras, since its C-terminal GAP domain catalyzes the hydrolysis of GTP-bound Ras to GDP-bound Ras. Subsequently, it has been shown that the N-terminal region of this large protein is essential to trigger downstream signals independently of its GAP activity, and acts as a positive effector of Ras [2]. N-terminal fragments of RasGAP, naturally generated by caspase cleavage, exert either an anti-apoptotic function (fragment N, 1–455) or a pro-apoptotic function (fragments N1, 1–157 and N2, 158–455) [3]. The N-terminal region of RasGAP (1–692) contains a pKC-conserved region 2 (C2) domain, a pleckstrin homology (PH) domain and a SH3 (Src Homology) domain flanked by two SH2 domains (Figure 1A). The SH2 domains mediate interactions with phospho-tyrosine containing proteins such as PDGF-R, EGF-R, v-Src, p62 and p190RhoGAP. The SH3 domain of RasGAP plays an essential role in Ras downstream signaling [2] and influences Rho-mediated cytoskeleton reorganization, independently from Ras [4]. The microinjection of a monoclonal antibody directed against RasGAP SH3 was shown to induce apoptosis in tumor cells but not in non-transformed cells [5]. A few RasGAP SH3-interacting proteins have been discovered so far. First studies have identified a GAP SH3-Binding Protein (G3BP) [6] and an unknown 14 kDa protein [7]. More recently, Aurora kinases have been shown to bind to RasGAP SH3 [8] and these interactions are suspected to play a crucial role in the Ras effector activity of RasGAP in cancer cells.

We reasoned that the use of specific peptidic ligands would enable a better mechanistic understanding of the role of RasGAP SH3 and of its interaction with Aurora kinases in Ras downstream signaling. The usual strategies to discover SH3 domain-targeting molecules appear ill-suited to RasGAP SH3, since this atypical domain has not been shown to interact with proline-rich sequences, which is a common feature of SH3 domains. An alternative approach was thus required.

Peptide aptamers are combinatorial recognition molecules that consist of a constant scaffold protein displaying a doubly-constrained variable peptidic region [9]. They bind to intracellular proteins with a high specificity and a strong affinity, and can thus modulate the function of their targets [10]. Their unbiased combinatorial nature allows interrogating the biological significance of numerous molecular surfaces on their cognate target proteins. The use of peptide aptamers can guide the discovery of small molecules targeting those molecular surfaces that are biologically validated and druggable [11].

Here, we report the selection and characterization of peptide aptamers that bind specifically to RasGAP SH3. One of these aptamers disrupts the interaction between RasGAP and Aurora B and induces caspase-independent tumor cell death.

## Results

### Selection of RasGAP SH3-interacting peptide aptamers

We used an improved yeast two-hybrid method and a new generation peptide aptamer library [12] to select peptide aptamers for their ability to bind to RasGAP SH3. We obtained 14 different peptide aptamers, whose binding specificity was determined using a yeast two-hybrid mating assay. As shown in Figure 1C, except for RG22 and RG24, no aptamer interacted with Nck or Grb2, two proteins containing respectively three and two SH3 domains that show sequence similarity with RasGAP SH3 (Figure 1B). RG22 and RG24 are two LexA-interacting aptamers, which exhibit an interaction phenotype with most LexA fusion proteins (albeit not with the LexA-Cdk2 control). Hence, we obtained 12 different peptide aptamers that interact specifically with RasGAP SH3. Except for RG18 and 25, all of them showed a comparable or weaker interaction phenotype with a LexA-SH2-SH3-SH2 bait construct. None of them showed an interaction phenotype with the full length RasGAP bait construct, whose apparent expression level in yeast was comparable to that of the other bait proteins presently used, as judged from the interaction phenotypes observed with the anti-LexA aptamers RG22 and RG24 (Figure 1C).

**Figure 2:**
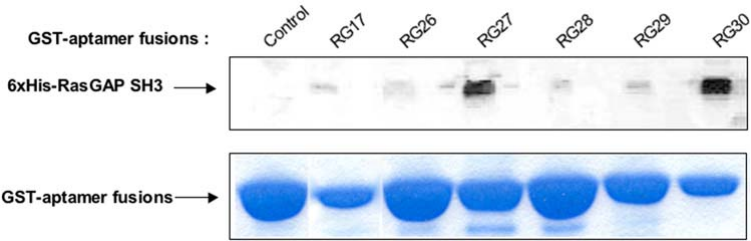
*in vitro* binding of peptide aptamers to RasGAP SH3. Pull-down assay with recombinant purified RasGAP SH3. Recombinant purified 6xHis-RasGAP SH3 was added to GST-peptide aptamer fusion proteins, coupled to glutathion-sepharose beads. “Control” is a peptide aptamer that was selected against another target protein. Captured protein was revealed with an anti-His antibody. GST-aptamer fusions coupled to beads were stained with Coomassie blue.

To confirm some of the yeast two-hybrid phenotypes, we performed pull-down experiments using 6 GST-RasGAP aptamer fusion proteins and recombinant purified 6xHis-RasGAP SH3. We confirmed the binding between RasGAP SH3 and all 6 peptide aptamers tested, with highly variable apparent binding affinities that were not determined by the amounts of GST-aptamer fusion proteins coupled to glutathione-sepharose beads and that did not match the intensities of the yeast two-hybrid phenotypes observed in the yeast two-hybrid mating assay (Figure 2).

### Mapping of the RasGAP SH3 peptide aptamer binding sites

**Figure 3:**
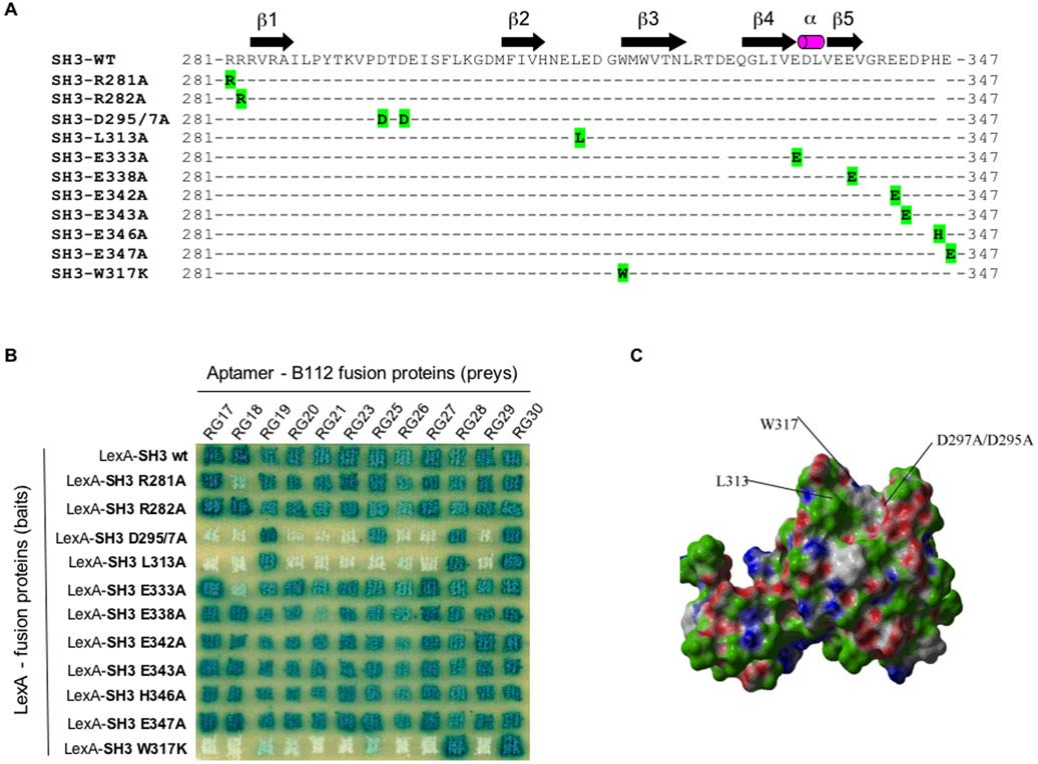
Mapping of the peptide aptamer binding sites on RasGAP SH3 by AptaPrint. (A) RasGAP SH3 aminoacid substitutions. (B) AptaPrint assay. 11 LexA fusions with RasGAP SH3 mutants were expressed in EGY48α yeast containing a 8 *lexAop-lacZ* reporter gene. Peptide aptamer-B112 fusions were expressed in EGY42a yeast. A yeast two-hybrid mating assay was performed. (C) NMR structure of the RasGAP SH3 domain. Residues W317, L313 and D295/7 are located.

We performed an AptaPrint assay to map the peptide aptamer binding sites on the RasGAP SH3 domain [11], [12]. Based on the NMR structure [13], we created 11 mutants to interrogate different molecular surfaces (Figure 3A). As shown in Figure 3B, our peptide aptamer collection showed different binding profiles against the RasGAP SH3 mutant panel, thus indicating that the aptamers bind to their target on different molecular surfaces. All aptamers (except RG28 and RG30) showed a decreased or abolished interaction phenotype against the W317K mutant, which suggests that this residue contributes to the integrity of most aptamer binding surfaces. Most aptamers (RG17, 18, 20, 21, 23, 26, 27, 29) failed to show an interaction phenotype when either L313 or D295/7 were mutated, which suggests that they bind to the pocket delineated by W317 and these three residues (Figure 3B and C). However, RG19 and RG25 seem to bind to a different surface as they retained their interaction phenotypes, either with the D295/7A and the L313A mutants (RG19), or with the D295/7A mutant (RG25). This AptaPrint did not provide information on the binding sites of RG28 and 30, since both aptamers retained their interaction phenotypes with all mutants.

### Peptide aptamer interactions in a mammalian cell context

**Figure 4:**
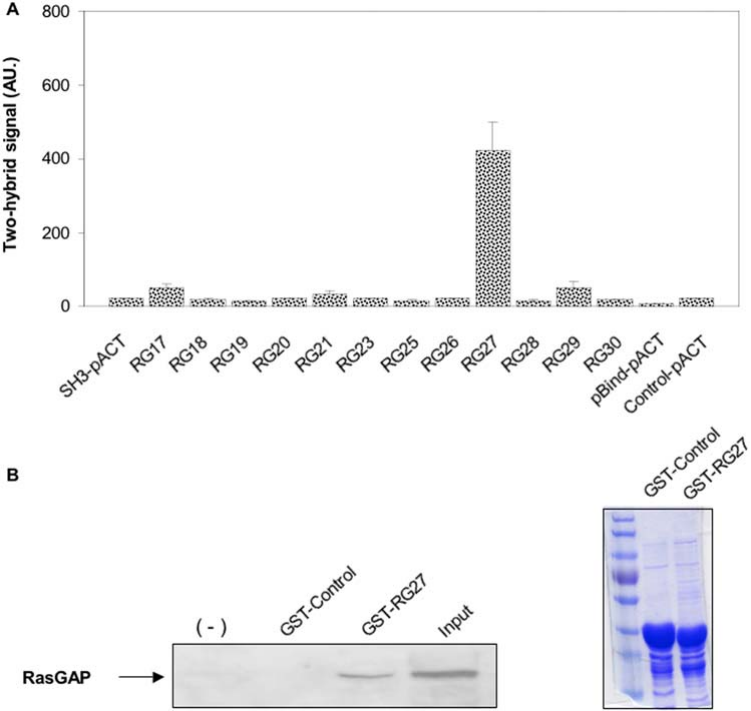
Peptide aptamer interactions in a mammalian cell context. (A) Mammalian cell two-hybrid assay. HeLa cells were co-transfected with a plasmid directing the expression of a GAL4-RasGAP SH3 bait protein, a plasmid containing a luciferase two-hybrid reporter gene, and plasmids directing the expression of VP16-peptide aptamer fusions. Luminescence was measured 48 h after transfection. Two independent experiments were performed. SH3-pACT: RasGAP bait with empty prey plasmid; pBIND-pACT: empty bait and prey plasmids; Control-pACT: empty prey plasmid only. (B) Pull-down assay from total protein extract. Left: A mouse liver total protein extract was added to glutathion sepharose beads, uncoated (−) or coated with GST-RG27 or GST-Control fusion proteins. Captured native RasGAP protein was revealed with a monoclonal antibody directed against the SH3 domain. Right: Coomassie staining of a SDS-PAGE revealing comparable amounts of GST-Control and GST-RG27 fusion proteins immobilized on glutathion sepharose beads.

We set out to determine the ability of the peptide aptamers to bind to the full length RasGAP protein in mammalian cells. To this end, we performed two-hybrid assays in HeLa cells that expressed a full-length RasGAP bait protein and all the selected aptamers, as preys. We failed to detect any significant interaction signal *(data not shown)*. We performed the same experiments using a RasGAP SH3 bait protein. RG27 was the only peptide aptamer that strongly interacted with RasGAP SH3 in this assay (Figure 4A). We then performed a pull-down experiment using a GST-RG27 fusion protein and a mouse liver total protein extract. We successfully captured the native endogenous RasGAP protein from the extract using the GST-RG27 fusion but not a GST-control aptamer fusion (Figure 4B). From all these observations, we conclude that aptamer RG27, at least, can interact with the native RasGAP protein in a mammalian cell context and that the lack of two-hybrid interaction phenotypes between the peptide aptamers and the full length RasGAP bait proteins is probably caused by an improper folding of the RasGAP moiety or, more probably, by a masking of the SH3 domain when the entire protein is expressed in yeast or human cell nuclei *(see* *Discussion* *)*.

### Peptide aptamer cytotoxic activity

**Figure 5:**
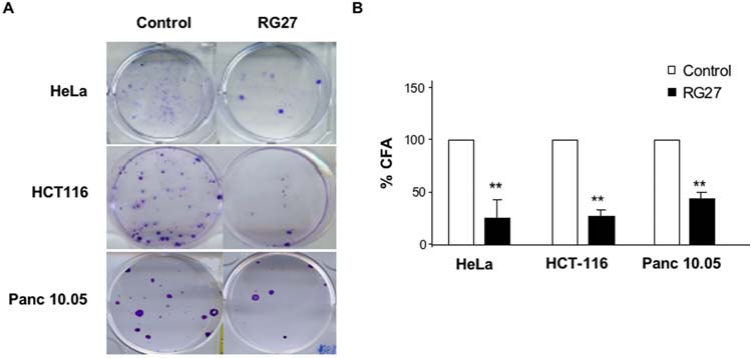
Cell growth inhibitory activity of RG27 peptide aptamer. (A) Colony formation assays. A control peptide aptamer or RG27 were stably expressed for two weeks in HeLa, HCT116 or Panc 10.05 cells. Cells were stained with crystal violet. (B) Quantification of results shown in (a) and in two other experiments **: p value <0.01.

We examined the ability of RG27 to inhibit tumor cell growth. We first performed colony formation assays on HeLa, HCT116 and Panc 10.05 cells, derived from cervix, colon and head-of-pancreas adenocarcinomas, respectively. HCT116 and Panc 10.05 cells bear a Ras oncogenic mutation. We stably expressed for two weeks a control aptamer (C6) or RG27. In all three cell lines, RG27 showed a growth inhibitory activity, in contrast with C6 that was inactive (Figure 5A and B).

**Figure 6:**
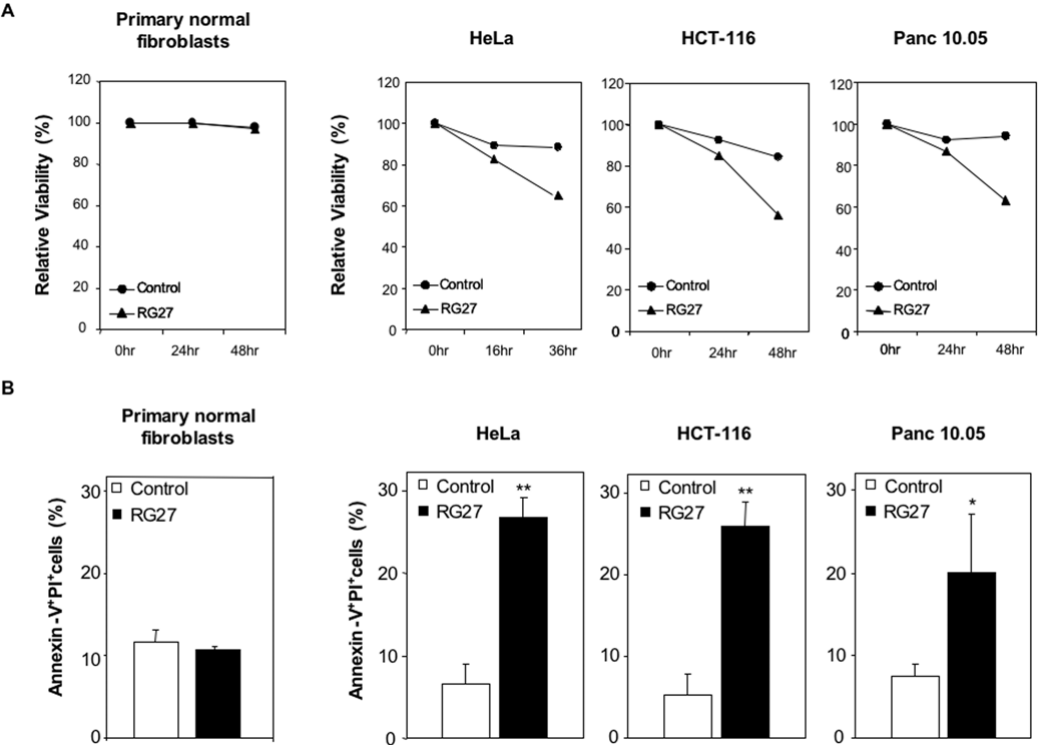
Cytotoxic activity of RG27 peptide aptamer. (A) Trypan blue exclusion assay. Control peptide aptamer (C6) and RG27 were transiently expressed in untransformed fibroblasts or in HeLa, HCT116 or Panc 10.05 tumor cell lines. The percentage of cells that excluded trypan blue was determined at different times after transfection. (B) Propidium iodide/Annexin V labeling assay. The percentage of cells that incorporated propidium iodide and that were positively labeled with Annexin V was determined by flow cytometry 36 h post-transfection for HeLa cells and 48 h post-transfection for HCT116 cells, Panc 10.05 cells and untransformed fibroblasts. Three independent experiments were performed in all assays. **: p value <0.01. Transfection rates were 75% for HeLa cells, 55% for HCT116, 40% for Panc 10.05 cells, and 70% for untransformed fibroblasts.

**Figure 7:**
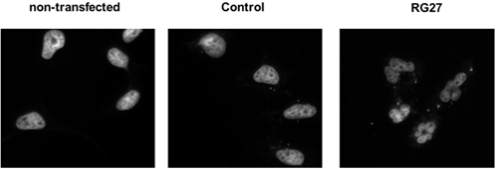
Nuclear fragmentation of RG27-expressing cells. The nuclei of HeLa cells untransfected or transfected with control aptamer or RG27 expression plasmids were labeled by DAPI 36 h post-transfection and observed using an epifluorescence microscope.

We then examined whether this inhibition of tumor cell growth was caused by an induction of cell death. We transiently expressed C6 or RG27 in the same cell lines as above. We evaluated cell viability at different times after transfection by performing a trypan blue exclusion test. In accordance with the results of the colony formation assays, RG27 but not C6 induced a marked decrease of the viability of tumor cell lines. Importantly, RG27 expression did not affect the viability of normal primary fibroblasts (Figure 6A). To further characterize RG27-induced cell death, we performed an Annexin V/propidium iodide double-labeling on the three tumor cell lines and on the untransformed fibroblasts. Consistent with the trypan blue exclusion test, RG27 but not C6 caused a significant increase in tumor cells labeled positively with these cell death markers, and remained inactive in untransformed fibroblasts (Figure 6B). We also examined the nuclei of HeLa cells expressing our peptide aptamers. In contrast to the nuclei of untransfected cells or of C6-expressing cells, the nuclei of cells expressing RG27 appeared clearly fragmented (Figure 7).

### Mechanism of action of cytotoxic peptide aptamer

**Figure 8:**
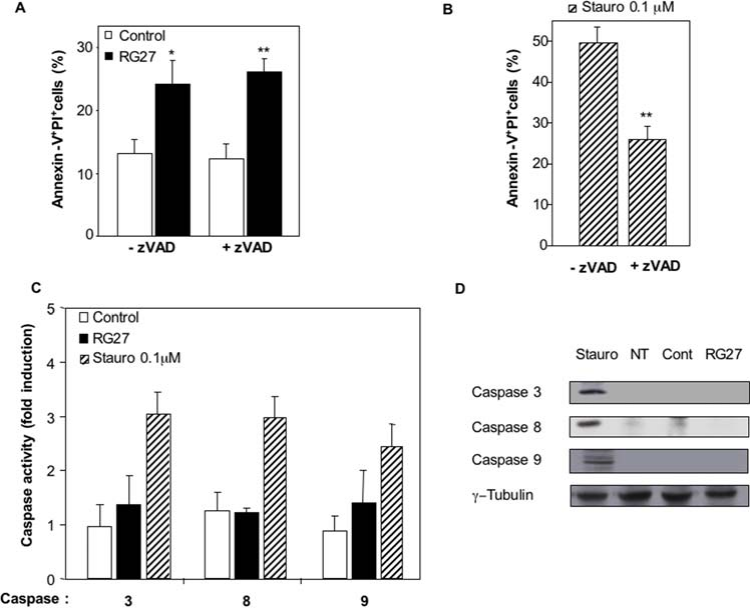
Caspase involvement in RG27-induced cell death. (A) Propidium iodide/Annexin V labeling assay in presence of caspase inibitor. The percentage of HeLa cells (transfected with control aptamer or RG27 expression plasmids) that incorporated propidium iodide and that were positively labeled with Annexin V was determined by flow cytometry 36 h post-transfection in presence or in absence of 50 µM pan-caspase inhibitor Z-VAD-fmk. (B) Same experiment as in (A), on HeLa cells treated with 0.1 µM Staurosporine for 36 h. (C) Caspase activity assays. Caspase 3, 8, 9 activities were measured by flow-cytometry in HeLa cells 36 h post-transfection or after 36 h of treatment with 0.1 µM Staurosporine. Values are expressed as “fold induction”, as compared to basal caspase activity values measured in non-transfected, untreated cells (D) Detection of active caspases. Western blots were performed from HeLa cell extracts obtained 36 h after transfection or treatment with 0.1 µM Staurosporine, using antibodies directed against active caspases. NT: non-transfected; Cont: control peptide aptamer.

To determine whether RG27-induced cell death involved caspases, we performed an Annexin V/propidium iodide double-labeling on transfected HeLa cells, in presence and in absence of the pan-caspase inhibitor Z-VAD-fmk [14]. As shown in Figure 8A, cell death induced by RG27 was not affected by Z-VAD-fmk, which inhibited Staurosporine-induced cell death, as expected (Figure 8B). In accordance with this result, we observed that RG27-expressing cells did not exhibit significant caspase 3, 8 and 9 activities, which could be detected upon Staurosporine treatment (Figure 8C). We also failed to detect active caspases in RG27-expressing cells (Figure 8D). Altogether, these observations establish that RG27 induces caspase-independent tumor cell death.

**Figure 9:**
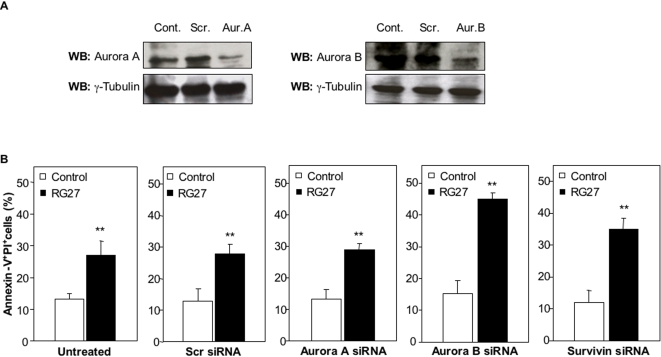
Aurora kinase and Survivin involvement in RG27-induced cell death. (A) Silencing of Aurora A and B expression. HeLa cells were transfected with scrambled (Scr) siRNAs or with siRNAs directed against Aurora A or B (Cont: untransfected cells). Aurora A and B expression was revealed by Western blot experiments using specific antibodies, and γ-tubulin expression was used as a control (B) HeLa cells were co-transfected with a plasmid directing the expression of either control aptamer C6 or RG27, and with siRNAs directed against Aurora A, Aurora B, or Survivin (“Untreated”: no siRNA; “Scr”: scrambled siRNA). Cells were labeled and analyzed as in Figure 6B.

We wondered whether RG27-induced cell death involved members of RasGAP SH3 interaction complexes suspected to play an important role in Ras downstream signaling [8]. We thus performed similar experiments on HeLa cells that we co-transfected with either C6- or RG27-expression plasmids and siRNAs against Aurora A, Aurora B, or Survivin. We first determined the efficacy of our Aurora siRNAs. As shown in Figure 9A, Aurora A and Aurora B siRNAs caused a comparable, partial expression knockdown of their cognate targets. Whereas Aurora A siRNAs did not affect RG27-induced cell death (as compared to a scrambled siRNA or no siRNA), Survivin siRNAs and even more so Aurora B siRNAs enhanced the cell death observed in RG27-expressing cells. This enhancement was caused by a potentiation of RG27 cytotoxic activity rather than by a simple additive phenomenon, since none of the tested siRNAs affected the basal cell death level observed in C6-expressing cells (Figure 9B).

**Figure 10:**
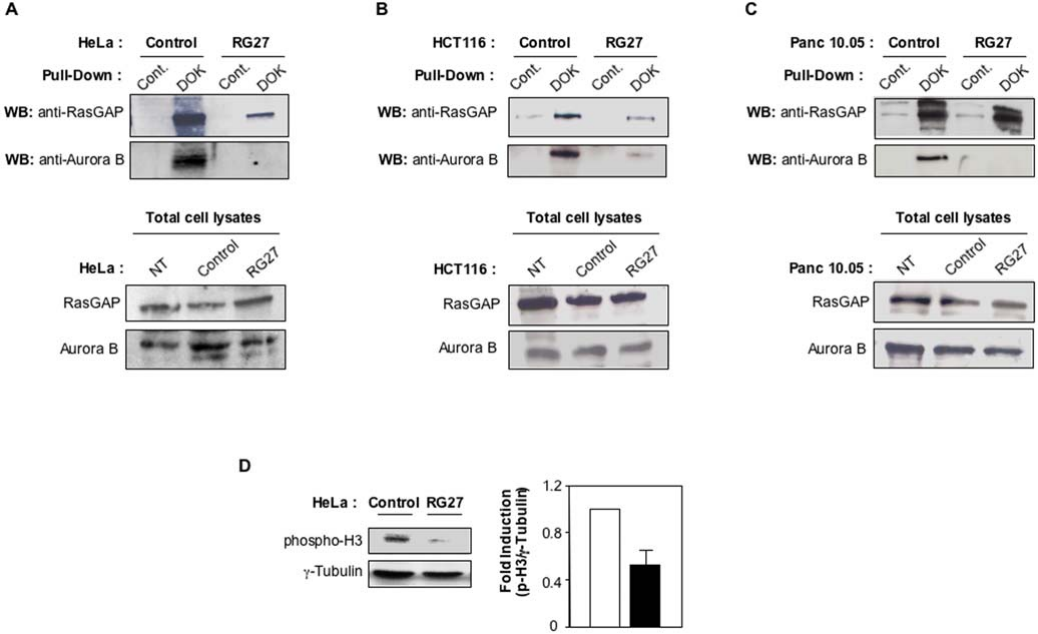
RG27-induced inhibition of RasGAP-Aurora B interaction and of Aurora B kinase activity. (A,B,C) RasGAP/Aurora B co-capture assay. Upper panels: A RasGAP pulldown assay was performed from HeLa cells (A), HCT116 cells (B) and Panc 10.05 cells (C) expressing control aptamer C6 or RG27, using either a RasGAP-SH2-interacting peptide (DOK) coupled to CNBr-sepharose beads or uncoupled beads (Cont.). Captured RasGAP and Aurora B proteins were revealed by Western blotting. Lower panels: Western blot experiments were performed from a fraction of the total HeLa, HCT116, and Panc 10.05 cell lysates used in the pulldown assays, and from lysates of non-transfected cells (NT), using RasGAP and Aurora B specific antibodies. (D) Aurora B kinase activity. The content of phosphorylated Histone H3 was determined by Western blot from soluble protein extracts of HeLa cells expressing either C6 or RG27 peptide aptamers. A Western blot using a γ-tubulin antibody was performed to obtain a loading control. Histogram shows the quantification of three independent experiments.

To further explore the mechanism of action of RG27, we examined the ability of this peptide aptamer to inhibit the interaction between RasGAP and Aurora B [8]. Using a RasGAP SH2-interacting peptide coupled to CNBr-sepharose beads, we performed a RasGAP pull-down assay from HeLa, HCT116 and Panc 10.05 cells expressing either the control peptide aptamer (C6) or RG27. As shown in Figure 10(A,B,C) we were unable to capture significant levels of Aurora B associated to RasGAP in RG27-expressing cells, in contrast to C6-expressing cells. This result was not caused by lowered expression levels of RasGAP or Aurora B in RG27-expressing cells. Interestingly, the amount of RasGAP captured from cells expressing RG27 was lower than that captured from cells expressing a control aptamer. This may be due to a steric hindrance caused by RG27 binding to the SH3 domain, which is situated just between the two SH2 domains that are bound by the Dok-derived peptide (Figure 1A).

Hence, RG27 disrupted RasGAP-Aurora B complexes in three different cell lines. We next examined the impact of this disruption on the kinase activity of Aurora B. To this end, we measured the phosphorylation levels of histone H3, a well-described substrate of Aurora B [15]. HeLa cells expressing RG27 showed a markedly decreased content in phosphorylated histone H3 as compared to C6-expressing cells (Figure 10D). This result indicates that RG27 expression induces a strong inhibition of Aurora B kinase activity.

## Discussion

We have used an improved peptide aptamer yeast two-hybrid method to select combinatorial peptidic ligands that interact specifically with RasGAP SH3. The sequence analysis of the peptide aptamer variable regions did not reveal any significant homology with naturally occurring proteins (Table 1). Interestingly, only one of the selected aptamers (RG26) harbored a variable region that contained a PxxP motif. Although more experiments would be needed to establish whether this motif mediates the interaction with RasGAP SH3, the fact that 11 out of the 12 selected peptide aptamers do not contain proline-rich variable regions validates our choice of a combinatorial approach to obtain peptidic ligands against this atypical SH3 domain.

**Table 1:** RasGAP SH3 peptide aptamer sequences

*Peptide Aptamers*	*Sequences of variable regions*
RG17	WYIRGKWVTGGRS
RG18	VWLACNRVCGSRG
RG19	TMHEKVWSPINSF
RG20	QHLTRGSPEFYHV
RG21	RNPNGCMPRYYHR
RG23	RPAKSACPTHPHL
RG25	CTWSYNAQGRIWV
RG26	ASTFPKPPRFYHV
RG27	KWVVSHARLMYSF
RG28	EGACPWEVRRLRF
RG29	RRLSRMCPSRYFT
RG30	GVAPWGTTTCRRF

None of the selected aptamers showed a two-hybrid interaction phenotype with a full-length RasGAP bait protein either in yeast or in HeLa cells. This result could be accounted for by the previously proposed masking of the SH3 domain by the two flanking SH2 domains, which, in absence of tyrosine-phosphorylated interacting partners such as p190-RhoGAP, limits the accessibility of the SH3 domain in the context of the full-length RasGAP protein only, but not in the context of a SH2-SH3-SH2 construct [7]. Yeast is known, indeed, to contain very few tyrosine kinases, and it is conceivable that the RasGAP SH2 tyrosine-phosphorylated interacting partners are absent from human cell nuclei or present in too little amounts. The fact that a RasGAP SH3 peptide aptamer affinity matrix could readily pull-down native RasGAP from a total protein extract supports the hypothesis of the masking of the SH3 domain in the two-hybrid setting. Surprisingly, RG27 was the only peptide aptamer that produced a strong two-hybrid interaction phenotype in HeLa cells. The weak or absent interaction phenotypes produced by all other peptide aptamers could be due to their lower steady-state expression levels and/or to the lower sensitivity of this assay as compared to the yeast assay.

The mapping of the peptide aptamer binding sites on RasGAP SH3 revealed different interesting facts. First, several molecular surfaces are targeted by this pool of 12 selected aptamers, as judged from the different profiles of yeast two-hybrid interaction phenotypes in the AptaPrint assay. Although the ability of large collections of peptide aptamers to “decorate” many surfaces on their cognate protein targets is constantly observed [11], it is striking that this holds true for a mid-sized collection of aptamers interacting with a small 60 aminoacid protein domain. Second, most peptide aptamers showed a decreased or abolished two-hybrid interaction phenotype with the RasGAP SH3 W317K mutant. Taken alone, this observation does not necessarily mean that most peptide aptamers establish a direct contact with W317. Indeed, since W317 seals together two spatially neighboring loops, the W317K mutation may induce long-range conformational changes on the SH3 domain. However, most peptide aptamers that showed a decreased or abolished interaction phenotype with the W317K mutant showed also a decreased or abolished interaction phenotype with the D295/297A and the L313A mutants. This observation strongly suggests that most peptide aptamers bind the pocket delineated by residues D295/7, L313 and W317, which may constitute a protein interaction hotspot.

We examined the bioactivity of RG27, an aptamer that targets the above-mentioned pocket, interacts with RasGAP SH3 in a HeLa cell two-hybrid assay, and interacts with the native full length RasGAP protein in a pull-down assay. In contrast to the C6 control, RG27 showed a marked cytotoxic activity when expressed in HeLa, HCT116 or Panc 10.05 tumor cell lines but not in normal primary fibroblasts. The lack of activity of RG27 in the latter cells is not due to a longer doubling time as compared to that of tumor cells (19–20 h for untransformed fibroblasts, Panc 10.05 and HCT116 cells; 16 h for HeLa cells). RG27-induced cell death is not inhibitable by the Z-VAD-fmk pan-caspase inhibitor, does not involve caspase 3, 8 and 9 activity, and can thus be qualified as caspase-independent cell death [14].

Among the few proteins known to form interaction complexes with the SH3 domain of RasGAP, Aurora kinases and Survivin are particularly compelling because of their involvement in the regulation of multiple cell cycle steps, in cell survival and in tumorigenesis [16], [17]. We showed that RG27-induced cell death was slightly potentiated by siRNAs directed against Survivin and, to a larger extent, by siRNAs directed against Aurora B. The lack of a detectable cytotoxic effect of our siRNAs on C6-expressing cells can be explained by their partial efficacy in knocking-down the expression of their cognate proteins and, in any case, by the fact that protein expression knockdown and protein targeting by specific ligands introduce different perturbations within regulatory networks, which give rise to different phenotypic responses [18]. These results suggested that RG27 might exert its cytotoxic activity by disrupting RasGAP-Aurora B interactions.

We showed that RasGAP-Aurora B complexes could not be detected in HeLa, HCT116 or Panc 10.05 cells expressing RG27. This peptide aptamer may disrupt RasGAP-Aurora complexes by competing directly with Aurora for binding to the SH3 domain of RasGAP. The lack of interaction between Aurora and a RasGAP W317K mutant supports this hypothesis [8]. Another disruption mechanism may involve an aptamer-mediated sequestration of RasGAP into aggresomes, according to a recently described phenomenon observed with the hepatitis B virus core protein and the human papillomavirus E6 protein [19]. However, several arguments speak against this hypothesis. First, we have performed immunofluorescence experiments in RG27-EGFP- expressing cells and we have not observed any perinuclear co-localization of RasGAP and RG27 *(data not shown)*, where aggresomes are located [19]. Second, the new-generation peptide aptamers presently used contain shorter variable regions (13 aminoacids instead of 20), which greatly enhances the average solubility level of recombinant purified aptamers. We would also expect an improved average solubility level of such peptide aptamers expressed in human cells. Third, we expressed untagged peptide aptamers in our cellular assays, which contrasts with the expression of much larger 6xHis-aptamer-VP22 fusion proteins by Tomai et al. (2006), which may be more prone to aggregation.

RG27-induced tumor cell death could be caused, at least in part, by the disruption of the interaction between RasGAP and the Aurora B-Survivin complex, and by the subsequent inhibition of Aurora B kinase activity. The antiproliferative and antitumoral properties of Aurora small molecule inhibitors are indeed well described [20], [21]. Since Aurora kinase activity was shown to be inhibited by the interaction with RasGAP in COS cells overexpressing Aurora [8], the inhibition observed in RG27-expressing cells might not be a direct consequence of the disruption of RasGAP-Aurora complexes and could be caused by a downstream event. Importantly, we cannot rule out that RG27-induced cell death is (also) caused by the disruption of another protein interaction that involves the SH3 domain of RasGAP. Our results are in line with the recent observation that a RasGAP SH3-derived minimal peptidic sequence sensitized tumor cell lines to a variety of cytotoxic molecules [22]. Although the mechanism of action of this peptide remains to be determined, it is likely to reside in the titration of RasGAP SH3 interacting partners (Aurora or another protein).

Ras is found mutated and/or deregulated in more than 30% of human cancers, and is thus considered as a highly interesting target to pursue for developing novel cancer therapies [23]. However, no Ras-targeting therapeutic molecule has passed clinical trials successfully up to date. The high intracellular concentration of GTP and the strong homology between numerous small G-proteins makes it difficult to discover Ras selective, potent inhibitors targeting the GTP-binding pocket. Farnesyl transferase inhibitors, which inhibit Ras function by preventing its translocation to the plasma membrane, have shown disappointing efficacy in clinical trials [24]. Molecules that disrupt protein interactions involving Ras are promising but still at early development stages [25]. An alternative Ras-targeting strategy consists of modulating the activity of the proteins that control the balance between the active and inactive forms of Ras, or that act as positive effectors of Ras. A seminal work using a monoclonal antibody against RasGAP SH3 has demonstrated that this domain was a potentially interesting target to pursue [5]. Our work further validates RasGAP SH3 (and more precisely the pocket delineated by residues D295/7, L313 and W317) as a promising therapeutic target in oncology. It points RG27 as a useful guide for the discovery of small molecule ligands against this target. To this end, we are now following two complementary approaches. First, ongoing NMR structural studies of complexes formed between RasGAP SH3 and RG27 or RG27-derived synthetic peptides will unveil precisely the molecular surface bound by the aptamer. This information may enable a structure-based drug design effort to obtain small molecule ligands to this surface. In parallel, high-throughput “AptaScreen” assays are performed to identify small molecules that disrupt the interaction between RasGAP SH3 and RG27 [11]. This dual strategy may yield promising small-molecule drug candidates.

## Materials and Methods

### Plasmid constructions

#### Bait plasmids

We amplified RasGAP SH3 (wild type and W317K mutant) coding sequences from existing plasmids [8] using oligonucleotides 5′-ATATGAATTCGGCGGTGAAGATAGAAGGCGTGTACG -3′ and 5′-TAATGGATCCTTATATTTTTCCTTCATGTGGATCTTC-3′ that contained an *Eco*RI and a *Bam*HI site, respectively. We ligated the PCR product into *Eco*RI/*Bam*HI-cut pEG202. We amplified the RasGAP SH2/3/2 coding sequence using oligonucleotides 5′-ATA TGAATTCGGTGGCGCCATACCGTTGACCGCTCCTCC-3′ and 5′- TAAT GTCGACCTATTGTTCTTGATCCTGCATTGGTAC-3′ that contained an *Eco*RI and a *Sal*I site, respectively. We ligated the PCR product into *Eco*RI/*Xho*I-cut pEG202.

To perform mutagenesis on RasGAP SH3, we used the following oligonucleotides: R281A-sens: 5′-GTGAAGATGCAAGGCGTGTACGAGCTATTCTACC-3′ R281A-rev: 5′-CTCGTACACGCCTTGCATCTTCACCGCCGAATTCCAG-3′; R282A-sens: 5′-GGTGAAGATAGAGCGCGTGTACGAGCTATTCTACC-3′; R282A-rev: 5′-CTCGTACACGCGCTCTATCTTCACCGCCGAATTCC-3′; D295/7A-sens: 5′-GCTATTCTACCTTACACTAAAGTACCAGCCACTGCTGAAATAAGTTTC-3′; D295/7A-rev: 5′-CATATCTCCTTTTAAGAAACTTATTTCAGCAGTGGCTGGTAC-3′; L313A-sens: 5′-GTTCATAATGAAGCAGAAGATGGATGGATGTGGGTTAC-3′; L313A-rev: 5′-CCATCCATCTTCTGCTTCATTATGAACAATGAACATATCTCC-3′; E333A-sens: 5′-CCTTATTGTTGCAGACCTAGTAGAAGAGGTGGG-3′; E333A-rev: 5′-CTCTTCTACTAGGTCTGCAACAATAAGGCCTTGTTCATCTGTTC-3′; E338A-sens: 5′-CTAGTAGAAGCGGTGGGCCGGGAAGAAGATC-3′; E338A-rev: 5′-GGCCCACCGCTTCTACTAGGTCTTCAACAATAAGGC-3′; E341A-sens: 5′-GGTGGGCGCGGAAGAAGATCCACATGAAGG-3′; E341A-rev: 5′-GATCTTCTTCCGCGCCCACCTCTTCTACTAGG-3′; E343A-sens: 5′-GCCGGGAAGCAGATCCACATGAAGGAAAAATATAAGG-3′; E343A-rev: 5′-CATGTGGATCTGCTTCCCGGCCCACCTC-3′; H346A-sens: 5′-GGAAGAAGATCCAGCTGAAGGAAAAATATAAGGATCCGTCGACC-3′; H346A-rev: 5′-CCTTATATTTTTCCTTCAGCTGGATCTTCTTCCCGGCC-3′; E347A-sens: 5′-GATCCACATGCAGGAAAAATATAAGGATCCGTCGACC-3′; E347A-rev: 5′-GATCC TTATATTTTTCCTGCATGTGGATCTTCTTCCCGG-3′. For each mutation, we performed two amplifications of the RasGAP SH3 coding sequence from the pEG202 construct, using on the one hand the cognate “sens” oligonucleotide and 5′- GTTGCCAGAAAATAGCGAGTTTAAACC-3′, and on the other hand the cognate “rev” oligonucleotide and 5′-GGAAAGAGTTACTCAAGAACAAGAATTTTCG-3′. We purified the PCR products and paired them according to their cognate mutant. We then performed a PCR amplification on each mix, adding at the end of the seventh cycle the oligonucleotides 5′-GTTGCCAGA AAATAGCGAGTTTAAACC-3′ and 5′-GGAAAGAGTTACTCAAGAACAAGAATTTTCG-3′. We co-transformed EGY48α yeast with the PCR products and *Eco*RI/*Xho*I-cut pEG202 to clone the RasGAP SH3 mutants by gap repair. We retrieved the plasmids by yeast DNA minipreps, transformed them into *E.coli*, and verified the mutations by sequencing.

#### Bacterial expression plasmids

We amplified peptide aptamer coding sequences from the library plasmids using the oligonucleotides 5′- CCACTTTAACTAATACTTTCAACATTTTCGG-3′ that hybridized to the GAL promoter and 5′-TGGGAACTCCTCGAGCTAGGCCAGGTTGGCGTCC-3′ that hybridized to the 3′ end of thioredoxin and that contained a stop codon. We ligated the PCR products into *Eco*RI/*Xho*I-cut pGEX4T1 (GE Healthcare).

#### Mammalian cell two-hybrid plasmids

We amplified the aptamer coding sequences from the library plasmids using the oligonucleotides 5′-TTATAGGATCCCTCGAGTTATGAGCGACAAGATCATCCACC-3′ and 5′-TGTCTGCTCGAAGCGGCCGCGGCTAGGCCAGGTTGGCGTCCAGGAACTCC-3′ that contained a *Xho*I and a *Not*I site, respectively. We ligated the PCR products into *Sal*I/*Not*I-cut pACT, a plasmid directing the expression of proteins fused to the VP16 activation domain (Promega). We amplified the RasGAP SH3 coding sequence from the pEG202 construct using the oligonucleotides 5′-TAAATA TAATGGATCCGCGACTGGCTGGAATTC-3′, which contained a *Bam*HI site, and 5′-GGAAAGAGTTACTCAAGAACAAGAATTTTCG-3′. We ligated the PCR product into *Bam*HI/*Not*I-cut pBIND, a plasmid directing the expression of proteins fused to the GAL4 DNA binding domain and the expression of a *Renilla* luciferase transfection marker (Promega).

#### Mammalian cell expression plasmids

We amplified the RG27 and the control aptamer coding sequences from the library plasmids using the oligonucleotides 5′-ATTCTAGCCTCGAGGAATTCACCATGAGCGACAAGATCATCC-3′and 5′- TGTCTGCTCGAAGCGGCCGCGGCTAGGCCAGGTTGGCGTCCAGGAACTCC-3′, that contained a *Xho*I and a *Not*I site, respectively. We ligated the PCR products into *Xho*I/*Not*I-cut pPER, a pCEP4-derived episomal vector that bears a CMV promoter and a hygromycin selection marker.

### siRNA sequences

Aurora A: 5′-AAAUGCCCUGUCUUACUGUCA-3′; Aurora B: 5′- AAGGUGAUGGAGAAUAGCAGU-3′; Survivin: 5′-AAGGCUGGGAGCCAGAUGACG-3′; Scrambled control: 5′- CAUGUCAUGUUCACAUCUCTT-3′. siRNAs were purchased from Sigma Proligo.

### Yeast two-hybrid selection of peptide aptamers

The selection was performed as described [12], using the yeast strains MB210/MB226 and the ACC02.2 peptide aptamer library [18]. We transformed MB210 with ACC02.2 and we obtained 2.8×10^7^ transformants. We transformed MB226 with pEG202-RasGAPSH3 and pSH18-34. We estimated the mating efficiency of MB210 at 84.6% and the number of diploid exconjugants at 3.3×10^8^. We plated 3.3×10^7^ diploids onto 20×9 cm plates with galactose/raffinose selective medium and incubated for 5 days. We replica-plated onto 20 X-gal galactose/raffinose medium. We picked 26 clones that grew in absence of leucine and adenine and that displayed a β-galactosidase activity. Library plasmids were recovered and re-transformed into EGY42a. The interaction phenotypes were confirmed by a mating assay with EGY191α transformed with pEG202-RasGAP SH3 and pSH18-34.

### Yeast two-hybrid interaction mating assays

The binding specificity and the AptaPrint assays were performed as described [12].

### Pull-down assays

#### RasGAP-aptamer interactions

We transformed pGEX4T1-aptamer plasmids into *E.coli* BL21 (DE3). We induced the expression of the GST-aptamer fusions with 1 mM IPTG at 20°C overnight. We collected the bacteria and resuspended them into lysis buffer I (50 mM Tris pH8, 0.1 M NaCl, 1 mM DTT) containing 1 mg/mL lysozyme. We froze and thawed the suspensions three times and sonicated on ice. We centrifuged the lysates at 13 000 g for 30 min, collected the soluble fractions, and analyzed aliquots on SDS-PAGE to verify that the respective amounts of GST-aptamer fusions were comparable. We thus immobilized equal amounts of GST-aptamer fusions on 100 µL glutathion sepharose 4B (GE Healthcare) at room temperature for 1 h. We washed the beads five times with 1 mL of lysis buffer.

We transformed pT7-7-RasGAP SH3 plasmid into *E.coli* C41 (DE3). We induced the expression of the 6xHis-RasGAP SH3 fusion with 1 mM IPTG at 20°C overnight. We prepared a soluble protein extract as described above. We purified the fusion protein using a Ni-NTA solid phase (Qiagen) according to the instructions of the manufacturer. To prepare a mouse liver total protein extract, we cut half a liver in small pieces to which we added 500 µL of lysis buffer II (40 mM Tris-HCl pH8, 500 mM NaCl, 1x Triton, 1 mM DTT, 15 mM EGTA, 1 mM Na_3_VO_4,_ and a Roche protease inhibitor coktail). We lysed the liver pieces with Fast Prep glass beads, running 2 cycles of 20 seconds in a FastPrep FP120 cell disrupter (Qbiogene). We centrifuged the lysate at 13000 g for 30 min at 4°C, collected the supernatant, and determined protein concentration using a Bradford assay (Sigma).

To the GST-aptamer solid phases, we added either 1.5 µg of recombinant purified 6xHis-RasGAP SH3 or 5 mg of total protein extract. We incubated 2 h at 4°C. We washed using lysis buffer I five times 1 mL and four times 5 mL, respectively. We boiled the beads in sample buffer and ran SDS-PAGE. We performed Western blot experiments using the mAb200 RasGAP antibody.

#### RasGAP-AuroraB interaction

We washed three times the transfected cells with ice-cold phosphate-buffered saline (PBS, pH 7.4) and we lyzed them for 15 min at 4°C in 250 µL a lysis buffer consisting of 50 mM HEPES pH 7.5, 150 mM NaCl, 1% Triton X-100, 10% glycerol, 1 mM MgCl_2_, 1 mM EGTA, 1 mM Na_3_VO_4_/10 mM NaF (phosphatase inhibitors) and a mix of protease inhibitors (1 mM PMSF, Complete TM protease inhibitors cocktail, Roche). We collected the lysates and centrifuged at 13000 g for 10 min at 4°C to remove insoluble materials. We determined protein concentrations using a Bradford assay (Sigma). We added 300 µg proteins to 100 µL of CNBr-activated sepharose bead slurries, uncoupled or coupled to a p62^DOCK^-derived peptide, and we incubated for 2 h at 4°C. We washed the beads three times in Hepes 50 mM pH7.5, NaCl 150 mM, Triton 0.1%, boiled them in sample buffer and ran a SDS-PAGE followed by a Western blot using the mAb200 RasGAP SH3 antibody (1∶1000) or a rabbit polyclonal Aurora B antibody (Abcam) (1∶2500). We used as secondary antibodies anti-rabbit or anti-mouse IgG coupled to horseradish peroxidase (GE Healthcare) (1∶10 000). We revealed the blots using a Supersignal system (Pierce).

### Determination of phosphorylated Histone H3 content

We lysed HeLa cells expressing either C6 or RG27 peptide aptamers and we extracted soluble protein contents as described above. We loaded 50 µg of soluble protein extracts onto a SDS-PAGE. We performed a Western blot using either a γ-tubulin antibody (Santa Cruz Sc-7396, 1∶1000) or a phospho-Serine 28 Histone H3 antibody (Abcam Ab5169, 1∶1000).

### Cell culture and cellular assays

#### Cell culture

Human cell lines HeLa, HCT116 and Panc 10.05 and normal fibroblasts (CRL 2068) were obtained from American Type Culture Collection (Rockville, MD) and maintained in DMEM or RPMI 1640 supplemented with 10% heat-inactivated fetal calf serum, 2 mM glutamine, 50 µg/mL of streptomycin, 50 µg/mL of penicillin and maintained in 5% CO2 at 37°C. We purchased media and supplements from Invitrogen.

#### Plasmid and siRNA transfections

For all plasmid transfections (except for the mammalian two-hybrid assays), we grew the cells in 6-well dishes to reach a 60% confluence. We incubated about 3×10^5^ cells with 2 µg of peptide aptamer expression plasmid and 5 µL Exgen-500 reagent (Euromedex, France) for HeLa and HCT116 cells or 2 µL Hiperfect (Qiagen) for Panc 10.05 cells. For plasmid/siRNA co-transfections of HeLa cells, we plated 2×10^5^ cells/well in 6-well plates. 12 h after plating, we incubated the cells with 2 µg of peptide aptamer expression plasmid, 20 nM of siRNA duplexes and 2 µL Hiperfect (Quiagen), according to manufacturer's instructions.

#### Colony formation assays

20 h after transfection, we collected and plated the cells onto multiple 60-mm plates. We cultured the cells for two weeks in presence of 0.1 mg/mL hygromycin. We fixed and stained the cells with a 0.1% crystal violet solution.

#### Trypan blue exclusion assay

We collected the transfected cells by trypsination at different times post-transfection. We added a trypan blue stain solution (0.4%, Gibco-Invitrogen) and we counted the number of unlabeled, viable cells. We calculated the relative viability as follows: relative viability (%) = (number of viable cell in treated group/number of viable cell in control group)×100. We counted at least 250 cells.

#### Propidium iodide and Annexin V labeling

At 36 or 48 h after transfection, we labeled the cells with Annexin V and propidium iodide according to the manufacturer's instructions (BD Biosciences). We analyzed the cells using a FACScalibur flow cytometer and the CellQuest software (Becton Dickinson). To test caspase involvement, we added or not 50 µM of Z-VAD-fmk caspase inhibitor (R&D Systems) immediately after transfection of HeLa cells. For a positive control, Staurosporine from Streptomyces (Sigma), kept in a stock solution of 10 mM in 100% DMSO, was added to HeLa cells at a final concentration of 0.1 µM for 36 h.

#### Nuclear labeling and observation

We grew HeLa cells on glass cover-slips. After 24 h, we transfected C6 or RG27 expression plasmid as described above. After 36 h, we fixed the cells for 15 min in 4% paraformaldehyde diluted in PBS. We permeabilized the cells in 0.5% Triton X-100 for 5 min, rinsed three times with PBS, incubated in a blocking solution (3% BSA in PBS) for 30 min, stained with DAPI diluted in blocking solution for 3 min, and rinsed twice with PBS. We observed the nuclei using a motorized epifluorescence microscope (Leica DM6000) endowed with a cooled CCD camera (Hamamatsu C5985).

#### Caspase activity assays

We performed fluorometric caspase activity assays on 10,000 cells by flow cytometry, according to the instructions of the manufacturer (R&D Systems, Abingon, UK). We detected active caspases by Western blots using 50 µg of soluble protein extracts from HeLa cells, and antibodies directed against active caspase 3, 8, and 9 (Biovision, USA) according to manufacturer's instructions. We used an antibody directed against γ-tubulin (Santa Cruz, USA) as a loading control. We used secondary antibodies coupled to horseradish peroxidase (GE Healthcare) and we revealed the blots using a supersignal system (Pierce).

#### HeLa cell two-hybrid assay

We seeded 10^4^ HeLa cells/well in a 96 well plate. We incubated overnight in triplicate 10 ng of pBIND-RasGAP SH3, 190 ng of pACT-aptamer, 100 ng of pG5*luc* (firefly luciferase reporter plasmid, Promega), 0.5 µL jetPEI (Qbiogene) and 9.5 µL NaCl 150 mM. We rinsed the cells and cultured them for another 24 h. We revealed sequentially the firefly (*luc*) and *Renilla* (*ruc*) luciferase activities using a Dual-Glo kit (Promega) and an Envision plate reader (Perkin Elmer). Since we observed no significant fluctuations in transfection rates (as judged from the homogenous *ruc* values), we directly used the *luc* values to quantify two-hybrid interaction phenotypes.
